# Analyses of the Xylem Sap Proteomes Identified Candidate *Fusarium virguliforme* Proteinacious Toxins

**DOI:** 10.1371/journal.pone.0093667

**Published:** 2014-05-20

**Authors:** Nilwala S. Abeysekara, Madan K. Bhattacharyya

**Affiliations:** 1 Department of Plant Pathology and Microbiology, Iowa State University, Ames, Iowa, United States of America; 2 Department of Agronomy, Iowa State University, Ames, Iowa, United States of America; Institute of Botany, Chinese Academy of Sciences, China

## Abstract

**Background:**

Sudden death syndrome (SDS) caused by the ascomycete fungus, *Fusarium virguliforme*, exhibits root necrosis and leaf scorch or foliar SDS. The pathogen has never been identified from the above ground diseased foliar tissues. Foliar SDS is believed to be caused by host selective toxins, including FvTox1, secreted by the fungus. This study investigated if the xylem sap of *F. virguliforme-*infected soybean plants contains secreted *F. virguliforme-*proteins, some of which could cause foliar SDS development.

**Results:**

Xylem sap samples were collected from five biological replications of *F. virguliforme*-infected and uninfected soybean plants under controlled conditions. We identified five *F. virguliforme* proteins from the xylem sap of the *F. virguliforme-*infected soybean plants by conducting LC-ESI-MS/MS analysis. These five proteins were also present in the excreted proteome of the pathogen in culture filtrates. One of these proteins showed high sequence identity to cerato-platanin, a phytotoxin produced by *Ceratocystis fimbriata* f. sp. *platani* to cause canker stain disease in the plane tree. Of over 500 soybean proteins identified in this study, 112 were present in at least 80% of the sap samples collected from *F. virguliforme*-infected and -uninfected control plants. We have identified four soybean defense proteins from the xylem sap of *F. virguliforme*-infected soybean plants. The data have been deposited to the ProteomeXchange with identifier PXD000873.

**Conclusion:**

This study confirms that a few *F. virguliforme* proteins travel through the xylem, some of which could be involved in foliar SDS development. We have identified five candidate proteinaceous toxins, one of which showed high similarity to a previously characterized phytotoxin. We have also shown the presence of four soybean defense proteins in the xylem sap of *F. virguliforme*-infected soybean plants. This study laid the foundation for studying the molecular basis of foliar SDS development in soybean and possible defense mechanisms that may be involved in conferring immunity against *F. virguliforme* and other soybean pathogens.

## Introduction

Sudden death syndrome (SDS) is an important soybean (*Glycine max* (L.) Merr) disease in US, Canada, Argentina, Brazil, Uruguay, Paraguay, and Bolivia [1], [2], [3]. In the United States, it is among the top four yield reducing soybean diseases [4]. In some states, SDS ranks second after soybean cyst nematode (SCN) in terms of yield suppression caused by these diseases in soybean [5]. It has been shown that soybean fields with high population density of SCN have a higher chance of SDS incidence [6]. The estimated soybean yield suppression from SDS in 2010 was 2.1% of the total yield valued at $0.82 billion [7].

Four *Fusarium* species, *Fusarium brasiliense*, *F. cuneirostrum*, *F. tucumaniae* sp. nov., and *F. virguliforme*, can cause sudden death syndrome across the world. All four species except *F. virguliforme* cause SDS in Brazil. *F. cuneirostrum* and *F. virguliforme* are causal agents of SDS in Argentina. *F. virguliforme* (Akoi, O’Donnell, Homma & Lattanzi), formally known as *F. solani* (Mart.) Sacc. f. sp. *glycines*, is the only *Fusarium* species that causes SDS in the U.S. [8], [9].

*Fusarium virguliforme* is a soil-borne fungus that belongs to the class Sordariomycetes and is known to produce one or more phytotoxins in culture media [10], [11], [12], [13]. Though the pathogen only infects soybean roots, the disease symptoms are seen on both roots and foliar tissues. The pathogen has never been isolated from the diseased foliar tissues. Hence, it is considered that toxin(s) produced by the fungus is responsible for the foliar SDS symptoms. It was suggested that in the presence of light, the phytotoxins secreted by the *F. virguliforme* to the culture media cause the degradation of the RuBisCo large subunit and the accumulation of free radicals, which presumably trigger programmed cell death leading to foliar SDS symptoms [14].

A purified 17 kDa proteinaceous toxin from the *F. virguliforme* cultures was shown to cause necrosis on soybean cotyledons and leaves [15]. However, the gene encoding this putative toxin has never been isolated. Recently, FvTox1 toxin was purified from the culture filtrates and the gene, *FvTox1*, encoding this toxin has been isolated [13]. The FvTox1 protein, expressed in an insect line, was shown to cause foliar SDS-like symptoms in soybean leaf discs, only in the presence of light [13]. FvTox1 is a 13.5 kDa acidic protein. This toxin can rapidly cause foliar SDS-like symptoms in leaf discs of soybean lines, highly susceptible to *F. virguliforme* [13]. Expression of a single chain variable fragment (scFv) antibody against FvTox1 enhanced foliar SDS resistance in transgenic soybean plants supporting the role of FvTox1 in foliar SDS development [16]. Investigation of *fvtox1* mutants suggested that FvTox1 is a major virulence factor involved in foliar SDS. The same study also revealed that additional toxins might play a minor role in foliar SDS development [17].

Proteomic research has gained new heights due to the availability of a wide array of gel-free proteomic technologies such as isobaric tagging for relative and absolute quantification (iTRAQ), multi-dimensional protein identification technology (MudPIT), isotope-coded affinity tag (ICAT), and coupled techniques such as liquid chromatography electrospray ionization tandem mass spectrometry (LC-ESI-MS/MS). Most of these techniques are faster, allow multiplexing of samples, and have better sensitivity and reproducibility [18]. Even with the currently available proteomic technologies, relatively few proteomic researches have been focused on the variation in the proteomes of host-pathogens interactions. These studies have shown that a variety of proteins, including peroxidases, chitinases, proteases, and pathogenicity related (PR) proteins to be differentially expressed in plants in response to pathogen invasion [19], [20], [21], [22]. Not only that there is variation in the relative abundance of certain proteins, some could also be induced only in response to either the compatible or the incompatible interaction [23], . These proteins could be involved in antifungal activities, signal transduction, anti-oxidation, protein folding, and an array of other plant functions and biological processes.

Recent studies have looked at the xylem sap proteome of several annual plants including soybean in detail [21], [29], [30], [31], [32], [33]. Very few studies have shown differential accumulation of proteins in xylem sap following pathogen infection [32], [24], [26]. Li et al [34] identified a stress-induced soybean protein in the stem exudates of soybean seedlings infected with *F. virguliforme*. Houterman et al. [24] reported the presence of 33 proteins including 21 tomato proteins and seven *F. oxysporum* proteins in the xylem sap of *F. oxysporum* infected tomato plants (*Solanum lycopersicum*). It is most likely that the host-selective proteinacious toxins produced by *F. virguliforme* are transported to the leaves via the vascular system to cause foliar SDS. Therefore, study of the xylem sap proteins of both infected and uninfected soybean plants could lead to identification of such *F. virguliforme* toxin proteins. The main objective of this study was to investigate if the xylem sap of *F. virguliforme-*infected soybean plants contains any secreted *F. virguliforme-*peptides/proteins. We applied LC-ESI-MS/MS in analyzing the proteomes of the xylem saps collected from either healthy, *F. virguliforme-*uninfected or *F. virguliforme-*infected soybean plants and identified five *F. virguliforme* proteins, one of which showed similarity to a previously characterized pathogen toxin.

## Materials and Methods

### Inoculum Preparation

*Fusarium virguliforme* isolates, Scott and Clinton, were grown on half strength potato dextrose agar (PDA) for about a week. Inoculum was prepared in sorghum meals as described by Hartman et al. [35]. In short, 200 g of sorghum (*Sorghum bicolor* (L.) Moench) seeds were soaked overnight in water in 1 quart Mason jars and autoclaved twice. Once the autoclaved sorghum seeds were cooled down, they were inoculated with 10 mycelial plugs containing conidial spores from each isolate. The cultures were allowed to grow on sorghum for four weeks; then harvested and air-dried. Fully dried inoculum was ground in a blender into powder. One part of the ground *F. virguliforme-*infested sorghum was mixed with 10 parts of sterile 1∶2 soil:sand mixture to make up the inoculum for the root infection assay.

### Plant Material and Xylem Sap Collection

The soybean variety “Spencer,” highly susceptible to *F. virguliforme,* was used in this study. Plants were grown using the modified layer method of Hartman and his co-workers [35], [36]. Three seeds were planted in one 8-oz styrofoam cup that were first half (150 mL) filled with the 1∶2 sterile soil:sand mixture and covered with 30 mL of the inoculum prepared on sorghum meals. Un-inoculated sterilized sorghum seeds were ground and mixed to 1∶10 ratio with the sterile 1∶2 soil:sand mixture to serve as a control. Cups were randomly placed in a growth chamber and the plants were grown at 25°C for 16 h under light (200 µ mol photons m^–2^s^–1^ light intensity) and at 16°C for 8 h in the dark. Plants were watered once daily. In each experiment, 120 plants were grown in either *F. virguliforme* inocula-containing soil or sorghum meal-mixed soil.

**Figure 1:**
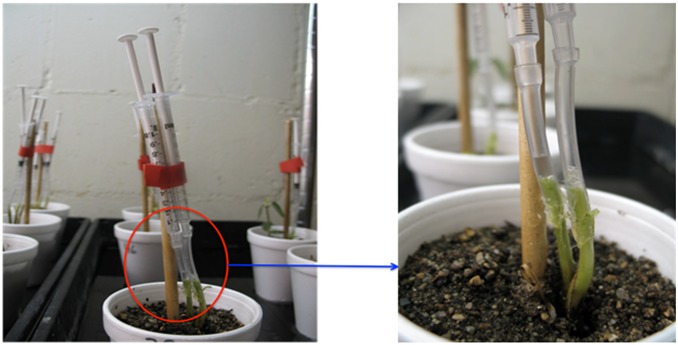
Collection of xylem sap from 14 to 21-day old *F. virguliforme-*infected or -uninfected soybean plants. The free end of a rubber tube attached to a 1 mL syringe was securely fasten to the cut soybean hypocotyl and sealed with Vaseline. Low pressure was created by pulling the plunger of the syringe to facilitate xylem sap accumulation.

Xylem sap was collected between 14–21 days, immediately following observation of foliar SDS symptoms. Plants were thoroughly watered in the evening of the day before collecting the xylem sap. A slightly modified method, described earlier by Djordjevic and his co-workers [31], was used to collect the xylem sap. The plants were de-capitated about 3–5 cm above the soil surface with a sterile surgical blade. Cut surface was gently wiped with a fresh lint-free Kimwipe (Kimberly-Clark Corporation; Roswell, GA) to avoid any contamination. The free end of a 5-cm long rubber tube attached to a 1 mL syringe was connected to the cut surface of the hypocotyl to collect the xylem sap. Vaseline was applied to establish a proper seal between the hypocotyl and the rubber tube. Syringe was securely tied to a small stick placed on the middle of the cup (Figure 1). Plunger of the syringe was pulled back to maintain a vacuum and to facilitate the xylem sap accumulation. Xylem sap was collected at a 2-h interval for up to 6 h from the tubing, and stored in pre-cooled 1.5 mL labeled Eppendorf tubes placed on ice. After each collection, Eppendorf tubes were stored at −80**°**C. Bradford assay (Bio-Rad, Hercules, CA) was conducted to estimate the concentration of proteins in the collected xylem sap. The xylem sap was collected from five independent experiments and stored at −80**°**C for analyses.

### 1D Gel Electrophoresis

Twenty µl of the crude xylem sap samples (containing about 1 µg of proteins) were fractionated in a 12% sodium dodecyl sulfate polyacrylamide gel (wt/vol) at 120 V for 90 min and stained with a mixer of 45% methanol (vol/vol), 10% glacial acetic acid (vol/vol), and 0.5% Coomassie brilliant blue G-250 (wt/vol).

### Protein Identification by Nano LC-ESI/MS/MS Analysis

Xylem sap samples were analyzed by nano LC-ESI/MS/MS at the Cornell University Proteomics and Mass Spectrometry Core Facility by Dr. Sheng Zhang and Mr. James McCardle. Ten xylem sap samples and one sample of the cell-free *F. virguliforme* culture filtrate (CF) were subjected to trypsin digestion followed by solid phase extraction (SPE). The tryptic digests were reconstituted in 2% acetonitrile (ACN) with 0.5% formic aid (FA) for nano LC-ESI-MS/MS analysis, which was carried out using an LTQ-Orbitrap Velos (Thermo-Fisher Scientific, San Jose, CA) mass spectrometer equipped with “Plug and Play” nano ion source device (CorSolutions LLC, Ithaca, NY). The Orbitrap was interfaced with an UltiMate3000 RSLC*nano* system (Dionex, Sunnyvale, CA). The nanoLC was carried out by a Dionex UltiMate 3000 RSLC*nano* system (Dionex, Sunnyvale, CA). The reconstituted peptides (2 µL) were injected under “User Defined Program” onto a PepMap C18 trap column (5 µm, 300 µm×5 mm, Dionex) at a 20 µL/min flow rate for on-line desalting and then separated on a PepMap C18 RP nano column (3 µm, 75 µm×15 cm, Dionex) which was installed in the “Plug and Play” device with a 10-µm spray emitter (New Objective, Woburn, MA) mounted in front of the Orbitrap orifice.

The peptides were eluted in a 90 min gradient of 5% to 38% ACN in 0.1% FA at 300 nL/min. The Orbitrap Velos was operated in the positive ion mode with nano spray voltage set at 1.5 kV and source temperature at 275°C. Internal calibration was performed using the background ion signal at m/z 445.120025 as a lock mass. The instrument was operated in data-dependent acquisition (DDA) mode using the FT mass analyzer for one survey MS scan at a resolution of 60,000 for precursor ions followed by MS/MS scans at a resolution of 7,500 on the top 10 most abundant peaks with multiple charged ions above a threshold ion count of 15,000 in the Linear Ion Trap mass analyzer. Dynamic exclusion parameters were set at repeat count 1 with a 30 s repeat duration, exclusion list size at 500, exclusion duration at 13 s, and mass width at ±10 *ppm* exclusion. Collision induced dissociation (CID) parameters were set at the following values: isolation width at 2.0 m/z, normalized collision energy at 35%, activation Q at 0.25, and activation time of 0.1 ms. All data are acquired under Xcalibur 2.1 operation software (Thermo-Fisher Scientific, San Jose, CA).

### Data Analysis

All raw data were searched using Mascot 2.2 (Matrix Science) software against the NCBI public database with taxonomy of Green Plants or the *F*. *virguliforme* genome sequence database containing 14,845 predicted *F. virguliforme* genes (http://fvgbrowse.agron.iastate.edu). The peptide tolerance was set to 10 ppm and MS/MS tolerance was set to 0.8 Da. Fixed carbamidomethyl modification of cysteine, variable modifications of methionine oxidation, deamination of asparagine and glutamine were considered. Data filtering parameters of 0.01 and 0.001 significance thresholds and an ion cut-off score of 32 and 27 k were applied to the results for searches against the Green Plants and *F. virguliforme* genome sequence databases.

False discovery rates (FDR) were calculated for each of the samples using the formula; FDR = (N_decoy_/N_real_+N_Decoy_)*100. This is an indication of the percentage of the random or “false” peptide identifications in the raw data. The relative abundance of the proteins identified by LC-ESI-MS/MS was estimated by determining the protein abundance index (PAI) and the exponentially modified protein abundance index (emPAI). Protein abundance index was calculated as follows: number of detected peptides divided by the number of observable peptides per protein normalized by the theoretical number of peptides expected *via in silico* digestion. The emPAI was calculated as 10^PAI^-1 [37].

### Functional Annotation

SignalP 4.0 server (http://www.cbs.dtu.dk/services/SignalP/) was used to identify N-terminal signal sequences [38]. Protein sequences obtained from NCBI were BLAST analyzed against the *Glycine max* protein database using Phytozome (www.phytozome.org). Sequences of the annotated soybean proteins were uploaded to Blast2Go V.2.6.2. program (http://www.blast2go.com/b2ghome) to perform functional annotation with gene ontology (GO) terms using default values [39]. Sequence distribution for molecular function was visualized at the ontology level 2 with a cutoff of 5. Interpro scan was also run and the results were merged with the annotations as described in the Blast2Go tutorial [40]. Sequences were also annotated using Kyoto Encyclopedia of genes and Genomes (KEGG) with the aid of Blast2Go to place the proteins into metabolic pathways.

## Results

### Similar Protein Profiles were Observed between Xylem Saps of *F. Virguliforme*-infected and -uninfected Plants Following 1D Gel Electrophoresis

**Figure 2:**
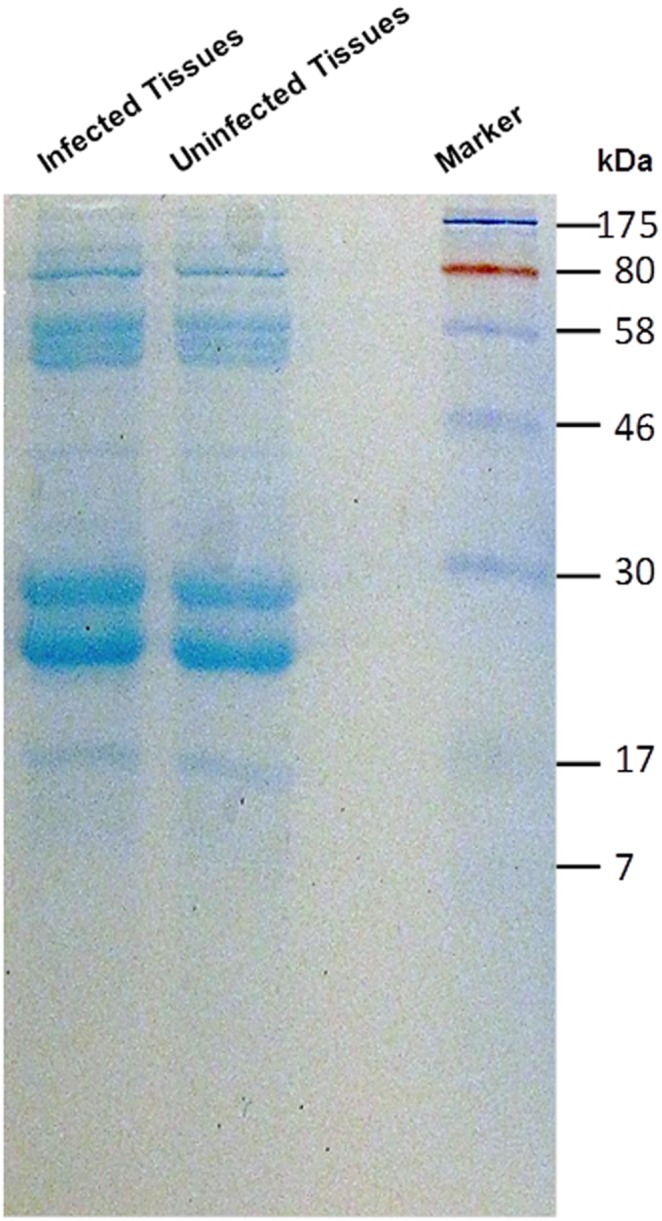
Protein profile of xylem sap collected from either *F. virguliforme-*infected or *F. virguliforme-*uninfected soybean plants. Xylem saps were fractionated by 12% SDS-PAGE gel and stained with Coomassie blue. Sizes of the molecular weight marker in kDa are shown at the right side. Infected, xylem sap from soybean plants infected with *F. virguliforme;* Uninfected, xylem sap from soybean plants that were not infected with *F. virguliforme.*

In order to identify proteins involved in foliar SDS, the protein profiles of xylem sap samples collected from the *F. virguliforme*-infected and -uninfected plants were compared by fractionating the sap samples on 12% SDS-PAGE gels. There were no qualitative or obvious quantitative differences between the crude xylem sap samples of the *F. virguliforme*-infected and -uninfected control plants for protein profiles (Figure 2).

### *F. virguliforme* Proteins were Identified from the Xylem Sap of the Infected Plants

Differences between xylem sap samples of the *F. virguliforme*-infected and -uninfected plants were detected through LC-ESI-MS/MS analysis. Five *F. virguliforme* proteins were identified in the xylem sap of the infected soybean plants. All five proteins possessed N-terminal secretory signal peptide sequences (Table 1). One of these proteins showed high sequence similarity to the cerato-platanin toxin, which was found to be the most abundant *F. virguliforme* protein in the xylem sap collected from the *F. virguliforme*-infected soybean plants (Table 1). Identified peptides of these five proteins are presented in Table S1.

**Table 1:** *F. virguliforme* peptides identified from the xylem sap of *F. virguliforme-*infected soybean plants.

Protein ID^a^	Description	Protein score	Protein mass (kDa)	# of peptides	Times identified^b^	emPAI^c^	Signal peptide
g3913	Cerato-platanin	6146	15.2	2	3(422)	1.2	Y
g12110	PAN_1 PAN domain	1008	28.2	2	2(72)	0.1	Y
g8948	Unknown	943	20.9	2	1(19)	0.6	Y
g11360	Unknown	687	25	2	2(45)	0.3	Y
g10227	Lipoprotein_15 Secreted repeat of unknown function	199	23.1	2	2(11)	1	Y

a Protein identification numbers are same as the gene IDs of the *F. virguliforme* genome database (http://fvgbrowse.agron.iastate.edu) [56].

b Number of times the peptides were identified from five biological replicates of xylem saps collected from *F. virguliforme*-infected soybean plants. The total number of times a peptide(s) was identified is presented in parentheses.

C Exponentially modified protein abundance index. This equals to 10^PAI^-1, which is proportional to the protein content in a protein mixture.

### Excreted Proteome of the Cell-free *F. virguliforme* Culture Filtrate Contains All Five Xylem Sap *F. virguliforme* Proteins

To determine if any of the five proteins (Table 1) identified from the xylem sap of the *F. virguliforme*-infected plants were also excreted to the culture medium, the proteomes of the cell-free *F. virguliforme* culture filtrates (CF) were investigated by conducting LC-ESI-MS/MS. Ninety four proteins were identified in the cell-free *F. virguliforme* culture filtrates (Table S2). Forty-four proteins with more than one peptide are presented in Table 2. A lipoprotein-15 was found to be the most abundant protein in the CF proteome. Of the 44 reported proteins, 37 shown to have predicted secretary signal peptides (Table 2). These proteins included cerato-platanin and FvTox1, with cerato-platanin toxin being the second most abundant protein (Table 2). All five proteins identified from the xylem sap of the *F. virguliforme*-infected soybean plants were also present in the pathogen CF (Table 2). The CF proteome contained proteins that showed high identity to cutinase, catalase, sporozite P67 surface antigen, eukaryotic type carbonic anhydrase, alpha amylase inhibitor, and laminin. Glycosyl hydrolases belonging to four different families were also identified (Table 2).

**Table 2:** Details of the *F. virguliforme* proteins identified in cell-free culture filtrates.

Protein ID^a^	Description	Protein score	Proteinmass (kDa)	# ofpeptides	Timesidentified^b^	emPAI^c^	Signalpeptide
g10227^d^	Lipoprotein_15Secreted repeat ofunknown function	11495	13.06	6	620	24.74	Y
g3913^d^	Cerato-platanin	2844	15.19	8	266	19.59	Y
g11360^d^	Unknown	2716	24.96	9	124	6.46	Y
g11634	Unknown	1899	19.99	7	76	3.74	Y
g12110^d^	PAN_1 PAN domain	1808	28.21	9	118	5.68	Y
g5600	Glycolipid anchoredsurface protein (GAS1)	1169	58.57	9	34	0.93	Y
g13851	Alpha amylase inhibitor	963	17.01	4	48	1.48	Y
g8948^d^	Unknown	880	20.85	2	23	0.35	Y
g12236	Unknown	774	15.60	8	69	4.85	Y
g11991	Glycosyl hydrolases family 16	673	29.25	7	35	0.91	Y
g7574	Cutinase	672	15.78	2	30	0.79	Y
g8691	TIL Trypsin Inhibitorlike cysteine rich domain	546	8.96	2	16	1.68	Y
g2004	Glycosyl hydrolasesfamily 15	543	78.02	8	26	0.51	Y
g6599	Unknown	506	15.87	3	12	1.17	Y
g4858	CFEM domain	432	23.13	3	30	1.58	N
g1353	Sporozoite P67surface antigen	419	21.89	2	9	0.33	N
g9768	Unknown	366	38.18	3	8	0.39	Y
g10536	CFEM domain	360	94.64	2	13	0.15	Y
g6924	FvTox1	346	40.75	2	20	0.37	Y
g13548	Glycosyl hydrolase family 4	312	40.11	4	32	0.17	Y
g2624	Catalase	263	78.29	3	15	0.23	Y
g11346	PLA2_B Lysophospholipasecatalytic domain	259	72.69	5	9	0.14	Y
g3867	Glycolipid anchoredsurface protein (GAS1)	252	49.54	3	6	0.21	Y
g10551	Glycosylhydrolases family 16	235	46.11	4	27	0.51	Y
g8790	Unknown	225	15.01	3	10	1.27	Y
g4057	Unknown	218	26.91	6	26	0.6	Y
g12686	Laminin_G_2 LamininG domain	208	29.63	4	9	0.53	Y
g4142	Unknown	207	18.56	3	17	0.95	Y
g7538	Isochorismatase	197	27.15	3	15	0.41	N
g1131	Unknown	189	32.68	2	5	0.21	Y
g3481	Squalene epoxidase	183	86.96	2	7	0.16	N
g7658	Cerato-platanin	164	23.72	2	4	0.3	Y
g2029	Glyceraldehyde 3-phosphatedehydrogenase,C-terminal domain	158	36.21	2	8	0.19	Y
g1554	Beta-glucosidase(SUN family)	136	47.22	2	3	0.14	Y
g7569	Eukaryotic-typecarbonic anhydrase	120	33.89	5	10	0.6	Y
g6537	Cis-muconatelactonizing enzyme	113	40.78	4	8	0.37	Y
g6259	Glycosyl hydrolasefamily 45	101	30.09	2	6	0.11	Y
g8786	Unknown	90	146.50	2	2	0.05	N
g5055	Ubiquitin Ubiquitin family	84	113.49	3	8	0.06	N
g8971	Chitosanase Fungalchitosanase	71	32.54	2	2	0.1	Y
g2888	N terminal extensionof bacteriophageendosialidase	68	87.69	2	3	0.08	Y
g2768	Unknown	51	15.18	2	2	0.5	Y
g8551	Trypan_PARP(Procyclic acidic repetitive protein)	50	69.16	2	5	0.1	Y
g12211	Peptidase family M28	40	38.73	2	2	0.18	N

a Protein identification numbers are same as the gene IDs of the *F. virguliforme* genome database (http://fvgbrowse.agron.iastate.edu) [56].

b Number of times a peptide(s) was identified in one biological replicate of the culture filtrate.

c Exponentially modified protein abundance index.

d *F. virguliforme* proteins that were identified from the xylem sap of *F. virguliforme*-infected soybean plants (Table 1).

### Soybean Proteins Detected in the Xylem Sap Samples Collected from the *F. virguliforme* Infected or Uninfected Plants

Over 500 soybean proteins were identified from the xylem saps collected from *F. virguliforme*-infected and -uninfected plants by conducting LC-ESI-MS/MS. The high number of protein detection could be due to the slightly high percentage of false discovery rate (FDR). The FDR percentage ranged from 2–3.3% among the different biological replicates. Details of the *Glycine max* accession numbers, GO annotations, as well as the biological replications from which these proteins were identified are provided (Table S3). Of these, 129 proteins were identified at least 80% of the time from sap samples of both *F. virguliforme-*infected and -uninfected plants (Table S4). However, at least two peptides were detected only for 112 soybean proteins (Table 3). Fifty of these proteins possessed predicted N-terminal secretory signal peptides. The most abundant proteins commonly found in the xylem saps of both *F. virguliforme-*infected and -uninfected plants were protease inhibitor/seed storage/lipid-transfer protein (LTP) family proteins (Table 3). Gamma-glutamyl hydrolase, 50S ribosomal proteins, trypsin and protease inhibitor protein, and peroxidases were some of the soybean proteins commonly found in sap samples of both *F. virguliforme-*infected and -uninfected plants.

**Table 3:** Soybean proteins identified from the xylem saps of both *F. virguliforme*-infected and -uninfected, healthy soybean plants.

Protein ID^a^	Description	Proteinscore	Proteinmass (kDa)	# ofpeptides	emPAI^b^	Signalpeptide
Glyma18g41320.1	Proteaseinhibitor/seed storage/LTP family	3281	13.0	7	134.77	Y
Glyma08g21410.1	50S ribosomal protein	2005	29.4	17	13.58	Y
Glyma07g01730.1	50S ribosomal protein	1684	29.2	20	13.87	Y
Glyma06g45700.1	Glycosyl hydrolase family 14	1161	55.8	20	2.17	N
Glyma13g34290.1	Gamma-glutamyl hydrolase	1073	37.8	13	3.52	Y
Glyma02g07140.1	Ribonuclease T2 family	630	27.0	8	2.6	Y
Glyma05g22180.1	Peroxidase	571	35.9	12	2.75	Y
Glyma08g45490.1	Trypsin andprotease inhibitor	506	22.0	10	5.33	Y
Glyma07g17000.2	Protease inhibitor/seedstorage/LTP family	492	11.1	6	4.07	Y
Glyma16g04240.1	Methionine synthase II(Cobalamine- independent)	480	84.4	23	0.84	N
Glyma17g17730.1	Peroxidase	479	36.0	11	2.75	Y
Glyma03g16620.3	Protease inhibitor/seed storage/LTP family	459	13.9	7	10.22	Y
Glyma12g06910.1	Heat shockprotein 70 kDa	445	75.6	11	0.67	N
Glyma12g19520.1	Malate dehydrogenase	389	36.4	11	1.01	N
Glyma03g32850.1	70 kDa heatshock protein	357	71.9	11	0.64	N
Glyma04g14650.1	Acyl CoAbinding protein	348	10.1	5	9.6	N
Glyma06g15030.1	Peroxidase	323	35.0	8	0.89	Y
Glyma16g04770.1	Nucleoside phosphatase	305	50.8	9	0.55	Y
Glyma19g37520.1	Enolase	305	48.0	12	0.82	N
Glyma02g00810.1	Cytosolic malatedehydrogenase	297	35.9	6	1.03	N
Glyma09g34770.1	Acyl CoA binding protein	293	10.1	5	9.6	N
Glyma18g52610.1	Cu/Zn superoxide dismutase	281	65.6	17	0.47	N
Glyma10g40720.1	Plant Basic Secretory Protein	280	26.9	6	0.79	Y
Glyma19g30140.1	Calmodulin	273	15.6	3	0.8	N
Glyma17g11790.1	Purple acidphosphatase-like protein	260	59.7	4	0.31	N
Glyma18g41590.1	Proteaseinhibitor/seed storage/LTP family	258	11.1	3	1.95	Y
Glyma05g28490.1	Glycine/serine hydroxymethyltransferase	256	53.3	8	0.43	N
Glyma08g11480.1	S-adenosylhomocysteine hydrolase	248	53.8	9	0.35	N
Glyma08g18760.1	TCP-1/cpn60 chaperonin family	248	53.8	7	0.35	N
Glyma03g04960.1	Proteaseinhibitor/seed storage/LTP family	239	12.9	6	4.21	Y
Glyma11g14950.1	70 kD heat shock protein	230	23.4	4	0.95	N
Glyma04g39475.1	Alginate lyase	228	24.6	5	0.66	Y
Glyma12g06606.1	ribulose-bisphosphatecarboxylase large chain	216	50.9	23	0.47	N
Glyma17g35720.1	Cysteine proteinase Cathepsin L	214	52.6	3	0.2	Y
Glyma13g16590.1	Peroxidase	213	36.1	8	1.02	Y
Glyma07g13710.1	Nucleosidediphosphate kinase	211	16.5	5	1.11	N
Glyma17g03350.1	Pathogenesis-relatedprotein Bet v I family	204	16.8	3	0.74	N
Glyma07g39120.1	Lactoylglutathione Lyase	196	31.7	2	0.1	N
Glyma02g09780.2	Proteaseinhibitor/seed storage/LTP family	195	10.4	5	4.58	Y
Glyma12g07780.3	Peroxidase	193	27.2	5	0.78	N
Glyma06g10650.2	Tyrosine3-monooxygenase/tryptophan 5-monooxygenaseactivation protein	192	29.3	8	0.91	N
Glyma08g45531.1	Trypsin and proteaseinhibitor	187	24.4	6	1.17	N
Glyma06g28890.1	Peroxidase	187	35.6	5	0.31	Y
Glyma15g19580.1	Cysteine proteinaseCathepsin L	178	39.7	4	0.38	Y
Glyma10g43990.1	Transketolase precursor	175	18.5	4	0.96	N
Glyma11g10240.1	Pollen allergen	175	52.1	4	0.42	Y
Glyma08g19180.1	Peroxidase	170	35.1	9	0.57	Y
Glyma05g38130.1	Thaumatin family	166	22.4	7	2.06	N
Glyma13g41960.1	Fructokinase	164	18.6	2	0.4	N
Glyma12g07370.1	Fasciclin domain	160	30.7	5	0.51	Y
Glyma16g08040.1	Zinc-binding oxidoreductase	158	34.6	8	0.9	N
Glyma14g35530.1	Plastocyanin-like domain	153	21.2	3	0.55	Y
Glyma07g00900.1	Lipoxygenase	151	96.9	9	0.26	N
Glyma01g32750.1	Proteaseinhibitor/seed storage/LTP family	144	12.2	3	2.45	Y
Glyma07g04960.1	Subtilisin/Kexin-RelatedSerine Protease	142	82.3	2	0.08	Y
Glyma04g04730.1	Subtilisin/Kexin-Related Serine Protease	142	82.3	3	0.08	Y
Glyma11g10480.2	Cyclophilin	141	18.4	5	0.57	N
Glyma06g47510.2	Ribosomal protein L16	138	21.0	2	0.35	N
Glyma10g13450.1	Lectin	134	30.2	6	0.69	Y
Glyma18g53150.1	Proteaseinhibitor/seed storage/LTP family	134	9.4	2	0.87	Y
Glyma04g02240.1	Plastocyanin	130	16.6	4	1.52	N
Glyma05g37490.1	Glycosylhydrolases family 28	128	26.7	3	0.6	N
Glyma05g35970.1	Profilin	125	14.1	2	0.24	N
Glyma03g40110.1	40S ribosomal protein S14	122	16.4	3	0.37	N
Glyma05g30380.1	Plastocyanin-like domain	115	13.2	4	2.98	Y
Glyma02g07150.1	Ribonuclease T2 family	115	27.1	7	2.19	Y
Glyma13g42330.1	Lipoxygenase	113	69.0	9	0.44	N
Glyma16g00410.1	Trypsin and protease inhibitor	112	56.0	4	0.12	N
Glyma12g08310.1	Mitochondrialchaperonin	110	52.9	6	0.17	N
Glyma10g07820.1	Monodehydroascorbate/ferredoxinreductase	109	43.7	5	0.24	N
Glyma02g05350.1	ferredoxin–NADP+ reductase [	109	40.6	3	0.17	N
Glyma04g14640.4	60S ribosomalprotein L13	108	23.8	2	0.14	N
Glyma13g17421.1	Glycosyltransferase	105	79.1	10	0.24	N
Glyma12g02790.2	Cyclophilin	104	18.4	2	0.66	N
Glyma08g41280.1	60S ribosomalprotein L34	102	13.8	2	0.56	N
Glyma19g42890.1	Hsp70 protein	102	62.0	4	0.14	N
Glyma06g02481.1	Subtilisin/Kexin-Related Serine Protease	100	82.2	6	0.17	Y
Glyma12g16340.1	Plastocyanin-likedomain	99	22.6	2	0.32	Y
Glyma15g04670.1	60S ribosomal protein L23A	98	17.3	2	0.71	N
Glyma15g04805.1	Histone H4	97	11.3	3	1.22	N
Glyma17g06090.1	Peroxidase	95	36.4	6	0.55	Y
Glyma02g18090.1	Lectin	94	22.6	6	0.74	Y
Glyma05g14330.2	Proteasomesubunit	93	24.2	4	0.22	N
Glyma04g01020.1	Fructose-biphosphatealdolase	92	14.1	2	0.54	N
Glyma16g17190.1	Pectin acetylesterase	91	43.9	2	0.16	Y
Glyma15g09530.1	GDSL-like Lipase/Acylhydrolase	91	43.1	8	0.81	Y
Glyma06g03410.1	Nad DependentEpimerase/Dehydratase	89	34.3	7	0.74	N
Glyma17g17850.1	aspartateaminotransferase	87	53.0	5	0.19	N
Glyma08g43690.1	40S ribosomal protein S8	86	25.1	3	0.28	N
Glyma10g28890.2	Calreticulin	81	48.3	5	0.3	Y
Glyma14g36850.1	Fructose-biphosphate aldolase	81	38.5	9	0.78	N
Glyma02g05370.1	40S ribosomal protein S4	73	29.8	4	0.37	N
Glyma08g21390.1	60S ribosomal protein L10	72	29.0	2	0.24	Y
Glyma11g21001.1	Dirigent-like protein	71	21.2	4	0.34	Y
Glyma11g13580.1	fructokinase	71	35.5	2	0.09	N
Glyma11g38220.1	PLAT/LH2 family protein	70	20.4	6	0.16	Y
Glyma16g01090.1	Subtilisin/Kexin-Related Serine Protease	70	86.6	2	0.08	N
Glyma14g10456.2	Glycosyl hydrolase family 10	70	109.9	3	0.12	N
Glyma17g33050.1	Demethylmenaquinone methyltransferase	70	59.9	5	0.08	Y
Glyma16g33710.1	Trypsin andprotease inhibitor	69	23.9	4	0.48	Y
Glyma13g39600.1	Serine carboxypeptidase	67	51.2	7	0.45	Y
Glyma03g23890.1	Zinc-binding dehydrogenase	67	38.1	6	0.09	N
Glyma06g18110.1	Glyceraldehyde 3-phosphate dehydrogenase	65	36.8	13	1.58	N
Glyma13g19830.1	GlutathioneS-transferase	65	27.1	2	0.26	N
Glyma12g31850.3	Carboxymethylenebutenolidase	63	21.9	2	0.23	N
Glyma18g47760.1	Hsp70 protein	58	28.9	2	0.17	N
Glyma08g45610.1	Trypsin andprotease inhibitor	52	26.2	3	0.43	Y
Glyma03g26060.1	Plastocyanin-likedomain	45	19.3	3	0.62	Y
Glyma20g26610.1	Plant basicsecretory protein	44	25.3	9	1.7	Y
Glyma18g44810.1	Cellulase(glycosyl hydrolase family 5)	42	52.8	5	0.27	Y
Glyma03g22260.1	Protein ofunknown function(DUF568)	38	25.7	2	0.28	Y
Glyma15g04290.1	Triosephosphateisomerase	33	27.4	7	0.77	N

a *Glycine max* protein identification number from Phytozome database (http://www.phytozome.net/search.php).

b Exponentially modified protein abundance index.

### Soybean Proteins that were Specific to the Xylem Sap Samples Collected from Plants, Either Infected or Uninfected with *F. virguliforme*

This study identified six soybean proteins that were only detected in xylem sap of *F. virguliforme*-infected plants; whereas, five soybean proteins only in healthy, *F. virguliforme*-uninfected plants. These proteins were detected in at least two out of the five biological replications of xylem sap samples collected from either *F. virguliforme-*infected or *F. virguliforme-*uninfected plants (Table 4 A, B). Only one infection induced proteins contained a predicted N-terminal secretory signal. Several stress related proteins were detected in the xylem sap of the infected plants but not in the sap of the uninfected plants. Three pathogenicity related proteins, glucan 1-3- β-glucosidase related protein, and NADP+ dependent malic enzyme were among the proteins identified from the infected plants (Table 4A). Pathogenicity related family 5 (PR5) protein was the most abundant of the six proteins identified from sap of the infected plants. Serine carboxypeptidase, disulfide oxidoreductase, and citrate synthase were among the proteins only detected in the sap of the healthy, *F. virguliforme-*uninfected soybean plants (Table 4B).

**Table 4:** Soybean proteins differentially accumulated in the *F. virguliforme*-infected (A) or *F. virguliforme*-uninfected (B) soybean plants.

Protein ID^a^	Description	Protein score	Protein Size (kDa)	# of peptides	Times identified^b^	emPAI^c^	Signal peptide
(A)							
Glyma11g10080.1	Glucan 1,3-beta-glucosidase-related	64	25.64	2	3	0.13	N
Glyma15g02230.1	NADP+-dependent malic enzyme	68	73.75	2	2	0.09	N
Glyma17g03340.1	Pathogenesis-related protein Bet v I family	109	17.19	3	3	0.43	N
Glyma17g04040.1	Plant invertase/pectin methylesterase inhibitor	66	19.88	2	2	0.17	Y
Glyma05g38110.1	pathogenesis-related thaumatin-like protein (PR5)	217	13.2	4	3	1.51	N
Glyma07g37240.2	Pathogenesis-related protein Bet v I family	315	1.09	4	4	1.09	N
(B)							
Glyma13g39730.1	Serine carboxypeptidases (lysosomal cathepsin A)	35	33.03	2	2	0.21	N
Glyma05g28480.2	Adenosylhomocysteinase	77	53.73	5	2	0.2	N
Glyma06g19820.1	Aldehyde dehydrogenase-related	50	55.39	2	2	0.12	N
Glyma08g02100.1	Disulfide oxidoreductase	112	52.42	2	3	0.13	N
Glyma15g15020.1	Citrate synthase	65	66.34	3	2	0.05	N

a *Glycine max* protein identification number from Phytozome database.

b Number of times the peptides were identified among five biological replications.

c Exponentially modified protein abundance index.

### GO Annotation and KEGG Pathway Analyses Revealed that Xylem Saps of Soybean Plants are Active in Carbon and Amino Acid Metabolisms

**Figure 3:**
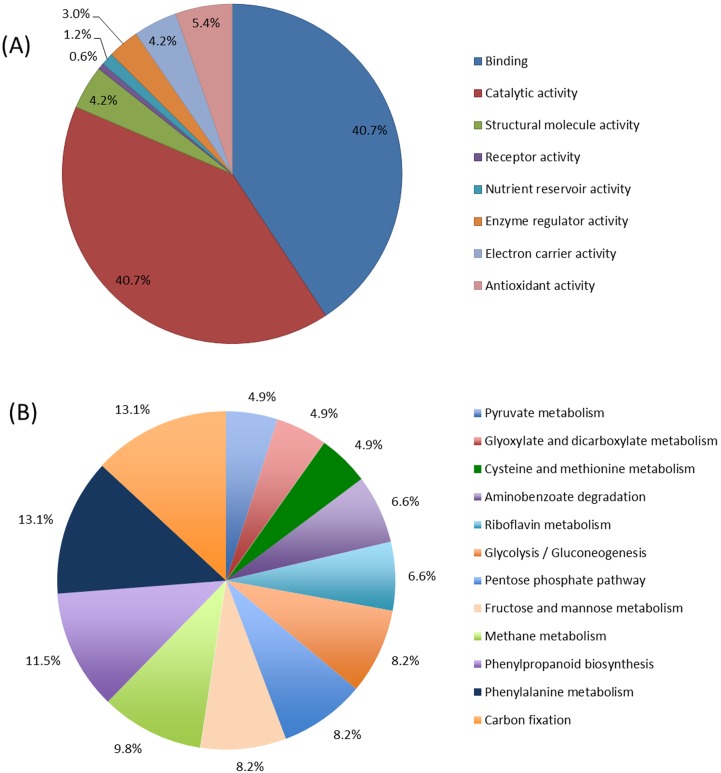
Classification of the 112 most abundant proteins identified from xylem saps of both *F. virguliforme-*infected and *F. virguliforme-*uninfected soybean plants based on molecular function. (A) Percentage of proteins in different functional categories at ontology level 2, with a cutoff of 5. (B) Secondary functional categories based on KEGG pathway. Only the prominent pathways with a sequence cutoff of 3 are reported here.

To determine the type metabolic activities in the soybean xylem saps, the sequences of the 112 putative soybean xylem sap proteins detected in xylem sap samples of both *F. virguliforme*-infected and *F. virguliforme*-uninfected plants were further subjected to functional annotation in GO terms. Most of these proteins were involved in more than one biological process and had more than one molecular function (Tables S5). Based on the sequence distribution with a cutoff of 5, majority of the 112 common xylem sap proteins showed binding and catalytic activities (Figure 3A). Among the 112 proteins that were assigned with enzyme commissions (EC), 44 metabolic pathways including phenylalanine metabolism, glycolysis, methane metabolism, carbon fixation, and phenylpropanoid biosynthesis were observed (Figure 3B, Table S6). The soybean xylem sap in general shown to be active in both carbon and amino acid metabolisms.

## Discussion

Sudden death syndrome is considered to be caused by one or more toxins released by *F. virguliforme* to the infected soybean roots because the root pathogen has never been detected in the above ground tissues showing foliar SDS. We have shown that FvTox1 is a major virulence factor that is involved in foliar SDS in soybean [13], [16], [17]. The foliar SDS was not completely absent in *fvtox1* infected soybean plants [17] suggesting that additional toxins may be involved in foliar SDS development; and it is conceivable that such toxins may be present in xylem sap of infected soybean plants. Therefore, the main objective of this investigation was to identify those possible *F. virguliforme* proteins by studying the xylem sap of *F. virguliforme*-infected soybean seedlings that showed foliar SDS. In addition, we investigated if there are changes in proteomes of xylem sap of soybean plants following *F. virguliforme* infection.

In this investigation we identified five *F. virguliforme* secreted proteins, including one with similarity to a known phytotoxin, cerato-platanin, from the xylem sap of infected soybean plants. This observation strengthens the previous speculations that the pathogen uses the vascular system to transport host-selective toxins to the above ground plant parts to cause foliar SDS. We, however, have to establish the functions of these putative proteinacious toxins by generating knockout mutants to establish their roles in generating foliar SDS in soybean.

We failed to detect FvTox1 from the xylem sap suggesting that our system is not sensitive enough to detect all proteins or peptides of the xylem sap. FvTox1 was localized to chloroplasts (H.K. Brar and M.K. Bhattacharyya, unpublished) and shown to be involved in SDS development in foliar tissues [16]. Most likely FvTox1 is produced at much lower concentration which was insufficient for detection in our LC-ESI-MS/MS study. This could also be the reason for not detecting the five *F. virguliforme* proteins in all five xylem sap replicates of the infected soybean plants. All five xylem sap *F. virguliforme* proteins were detected in the CF proteome suggesting that they all have functional signal peptides for excretion; and most likely they were excreted by the pathogen into the infected roots for uploading into the xylem vessels.

LC-ESI-MS/MS used in this study does not detect peptides that are smaller than seven amino acids. Therefore, if there were any small nonribosomal phytotoxic peptides in the xylem sap, then those were not identified in our study.

Cerato-platanin is a phytotoxin produced by the ascomycete fungus *Ceratocystis fimbriata* f. sp. *platani* that causes canker stain disease in the European plane tree (*Platanus acerifolia*). This 12.4 kDa proteinaceous toxin, the first member of the cerato-platanin protein family, was identified from the culture filtrate of *C. fimbriata* f. sp. *platani* [41], [42]. Cerato-platanin toxin is considered to be a pathogen associated molecular pattern (PAMP) because it can induce defense related responses in the host plant [43]. Proteins belonging to the cerato-platanin protein family can enhance plant defenses by inducing defense related genes, phytoalexins synthesis, and initiating cell death [43], [44], [45], [46]. We identified a cerato-platanin-like protein from both xylem sap and *F. virguliforme* (*Fv*) culture filtrates. Investigation of mutants for the gene encoding this cerato-platanin-like protein will assist us in determining the function of this protein in *F. virguliforme.*

This xylem sap study, in addition to identifying candidate pathogenicity *F. virguliforme* proteins, detected pathogenicity related soybean protein families (Table 4A). Beta-1,3-glucanase is a pathogenic related family 2 (PR-2) type protein known to be secreted upon pathogen attack and shown to be present in the xylem sap of pathogen infected plants [26], [47],[48]. This protein has the ability to inhibit the fungal growth by degrading β-1,3-glucans of the fungal cell wall [49], [50]. Cytosolic isoforms of NADP malic enzyme are known to be involved in plant defense responses [51], and an NADP-dependent malic enzyme was detected in the xylem sap of *F. virguliforme-*infected soybean plants (Table 4A). The expression of pathogenicity-related Bet v I family proteins (PR10) are known to be induced during wounding, abiotic stress, or pathogen infection. The possible roles of these proteins in the soybean- *F. virguliforme* interaction are yet to be established.

Most of the soybean proteins identified in this study were previously reported in the xylem sap of other plant species including soybean [31], [32], [33]. Xylem saps have been shown to contain peroxidases, which may play a role in plugging damaged vascular tissue [52]. We observed peroxidases in the xylem saps of both infected and healthy plants. Usually peroxidases are produced in response to biotic stresses; but some peroxidases are not specific to infection. Even though found in the xylem sap, the origin of these proteins is still questionable. The xylem sap collection method could impose stress on the plants. Detection of stress-induced proteins such as ribonucleases from both diseased and healthy plants could therefore have resulted from the stress associated with the xylem sap collection method.

Ligat et al. [53] has shown that xylem sap proteins can be produced in the root tips and then loaded into the xylem sap. Other studies have also suggested these proteins to be synthesized in the roots [29], [52], [54]. Hence it is possible the xylem sap proteins identified in this study are in fact secreted by the root tissues and loaded into the xylem sap. Some of these sap soybean proteins could have been synthesized to defend *F. virguliforme* infection. Pathogenicity related (PR) proteins are implicated as plant defense molecules and were identified in the xylem sap of *F. virguliforme*-infected plants. Further study is needed to determine if PR genes are transported long distance as a defense arsenal and involved in defending soybean against *F. virguliforme* and other pathogens.

## Conclusion

This study identified five secreted *F. virguliforme* proteins from the xylem sap of soybean plants infected with *F. virguliforme*. These proteins were also found to be secreted by *F. virguliforme* into the culture medium. This study provides evidence that *F. virguliforme* secreted proteins travel through the xylem sap. The presence of a protein with high similarity to the phytotoxin, cerato-platanin in xylem sap of the *F. virguliforme*-infected plants shows that multiple host-selective toxins are produced by *F. virguliforme* and could be responsible for the foliar SDS development. We have also detected 112 soybean proteins in xylem saps of at least eight of the 10 replications. Most importantly we have identified four types of pathogenicity-related defense proteins only from the xylem sap of *F. virguliforme*-infected soybean plants. Thus, this study laid the foundation for studying the molecular basis of foliar SDS development in soybean and possible defense mechanisms that may be involved in conferring immunity against *F. virguliforme* and other soybean pathogens.

The mass spectrometry proteomics data have been deposited to the ProteomeXchange Consortium (http://proteomecentral.proteomexchange.org) via the PRIDE partner repository [55] with the dataset identifier PXD000873.

## Supporting Information

Table S1 **Peptides of five** ***F. virguliforme*** **proteins that were detected in the xylem sap of the infected soybean plants.**
(XLSX)

Table S2 **Peptides of** ***F. virguliforme*** **proteins that are excreted to the culture medium. First five proteins were identified in xylem sap of the** ***F. virguliforme*** **-infected plants.**
(XLSX)

Table S3 **Gene Ontology (GO) annotation of over 500 soybean proteins identified from the xylem sap.**
(XLSX)

Table S4 **Detection of over 500 soybean proteins across 10 biological replications of xylem sap: (i) five replications of** ***F. virguliforme*** **-uninfected tissues and (ii) five replications of** ***F. virguliforme*** **-infected tissues. 0, not detected; 1, detected.**
(XLSX)

Table S5 **Blast2Go results for the 112 soybean proteins identified in the xylem saps of both** ***F. virguliforme*** **-infected and** ***F. virguliforme*** **-uninfected soybean plants.**
(XLSX)

Table S6 **Analyses for KEGG pathway of the 112 most abundant soybean proteins identified from both** ***F. virguliforme*** **-infected and** ***F. virguliforme*** **-uninfected soybean plants.**
(XLSX)
